# CLICK: one-step generation of conditional knockout mice

**DOI:** 10.1186/s12864-018-4713-y

**Published:** 2018-05-02

**Authors:** Yoshiki Miyasaka, Yoshihiro Uno, Kazuto Yoshimi, Yayoi Kunihiro, Takuji Yoshimura, Tomohiro Tanaka, Harumi Ishikubo, Yuichi Hiraoka, Norihiko Takemoto, Takao Tanaka, Yoshihiro Ooguchi, Paul Skehel, Tomomi Aida, Junji Takeda, Tomoji Mashimo

**Affiliations:** 1 Institute of Experimental Animal Sciences, Graduate School of Medicine, Osaka University, Osaka, 565-0871 Japan; 2 Genome Editing Research and Development (R&D) Center, Graduate School of Medicine, Osaka University, Osaka, 565-0871 Japan; 3 Mouse Genomics Resource Laboratory, National Institute of Genetics, Shizuoka, 411-8540 Japan; 4 Medical Innovation Center, Graduate School of Medicine, Kyoto University, Kyoto, 606-8507 Japan; 5 Laboratory of Recombinant Animals, Medical Research Institute (MRI), Tokyo Medical and Dental University (TMDU), Chiyoda, Tokyo, 101-0062 Japan; 6 Laboratory of Molecular Neuroscience, MRI, TMDU, Tokyo, 113-8510 Japan; 7 Department of Otorhinolaryngology-Head and Neck Surgery, Graduate School of Medicine, Osaka University, Osaka, 565-0871 Japan; 8 KAC Co., Ltd., Kyoto, 604-8423 Japan; 9 Centre for Integrative Physiology, University of Edinburgh, Edinburgh, EH8 9XD UK; 10 Present address: McGovern Institute for Brain Research, Massachusetts Institute of Technology, Cambridge, MA 02139 USA; 11 Department of Genome Biology, Graduate School of Medicine, Osaka University, Osaka, 565-0871 Japan

**Keywords:** CRISPR/Cas9, CLICK, Zygote electroporation, Long single-stranded DNA, Cre-loxP system

## Abstract

**Background:**

CRISPR/Cas9 enables the targeting of genes in zygotes; however, efficient approaches to create loxP-flanked (floxed) alleles remain elusive.

**Results:**

Here, we show that the electroporation of Cas9, two gRNAs, and long single-stranded DNA (lssDNA) into zygotes, termed CLICK (CRISPR with lssDNA inducing conditional knockout alleles), enables the quick generation of floxed alleles in mice and rats.

**Conclusions:**

The high efficiency of CLICK provides homozygous knock-ins in oocytes carrying tissue-specific Cre, which allows the one-step generation of conditional knockouts in founder (F0) mice.

**Electronic supplementary material:**

The online version of this article (10.1186/s12864-018-4713-y) contains supplementary material, which is available to authorized users.

## Background

The Cre/loxP system is one of the most valuable tools for genome engineering. In this system, Cre recombinase efficiently catalyzes recombination between two 34-bp consensus loxP sequences in any cellular environment, enabling the conditional transgenesis or knockout of genes in mice to study gene functions in specific tissues or at specific time points during development [1, 2]. Within ten years, the high-throughput generation of conditional alleles for virtually all mouse genes in embryonic stem (ES) cells has been achieved by the International Knockout Mouse Consortium [3, 4]. However, more than 10% of mouse ES genes have still not been modified because of targeting problems. Furthermore, the substitution of alternative exons or specific DNA fragments, such as promoters or enhancers, requires the generation of new targeting alleles.

Recently, the clustered regularly interspaced short palindromic repeats (CRISPR)/CRISPR associated (Cas) system has enabled the knockout of genes in zygotes via non-homologous end-joining (NHEJ) with unprecedented simplicity and speed [5–7]. Regarding conditional alleles, targeted insertions of two loxP sites have been generated by genome editing tools with two loxP-containing single-stranded oligodeoxynucleotides (ssODNs) [8, 9] or with a single double-stranded DNA template, containing flanking homology arms (HA) via homology-directed repair (HDR) [6, 10]. However, the efficiency of these methods is not optimal in zygotes because of the lower rate of HDR compared with NHEJ. In addition, when only one of two loxP sites, or two loxP in *trans* are inserted at the targeted site, it is very difficult to obtain recombination between the two sites by further crossing. In this study, we have developed a one-step generation method for floxed mice using the CRISPR/Cas system with a long single-stranded DNA (lssDNA) composed of a targeted exon flanked by two loxP sites, which was constructed using a simple method using nicking endonucleases as we previously reported [11] (Additional file 1: Figure S1).

## Results

### Microinjection of CRISPR/Cas9 with lssDNA to generate a floxed allele

**Fig. 1:**
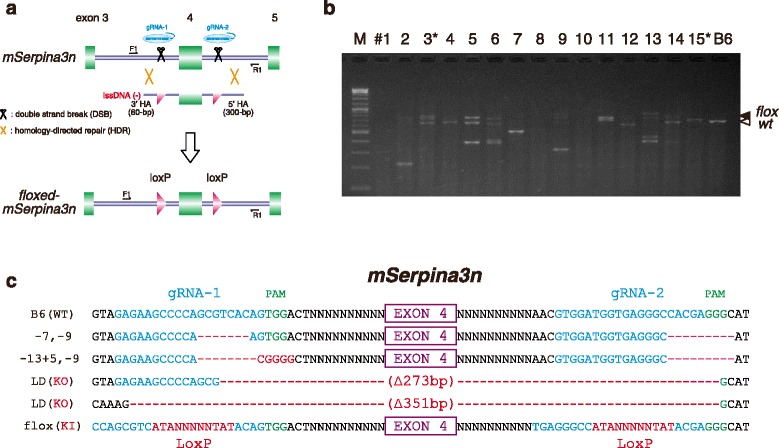
Mouse floxed alleles generated by microinjection of two gRNA, Cas9 mRNA and lssDNA into zygotes. **a** An approach to generate *Serpina3n* floxed alleles using the CRISPR/Cas system with long single-stranded DNA (lssDNA) composed of the targeted exon flanked by two loxP sites (Additional file 2: Figure S2). **b** PCR analysis of representative delivered mouse pups (#1–15) showing different types of mutations, indels, LD, and floxed alleles (black arrow) at the targeted *Serpina3n* locus. The primer sets (‘small’ in Additional file 2: Figure S2) were used for PCR and sequence analysis. Asterisks indicate pups used for testing germline transmission (Additional file 15: Table S1). M: 100 bp DNA ladder marker. **c** Representative examples of the targeted *Serpina3n* loci generated by the microinjection of two gRNA (gRNA-1 and gRNA-2), Cas9 mRNA and lssDNA into B6 mouse zygotes (Additional file 3: Figure S3)

The proof of concept experiment started with the design of two gRNAs targeting introns on either side of exon 4 of the serine peptidase inhibitor clade A member 3 N (*Serpina3n*) gene, and lssDNA, which contains exon 4 flanked by two loxP sequences with a 60-bp 3′ HA and an extended 300-bp 5′ HA to avoid the effect of possible 5′ exonuclease activity, as previously described [11] (Fig. 1a and Additional file 2: Figure S2). To increase Cas9 endonuclease activity, we also used Cas9-polyA plasmids (RIKEN BRC: RDB13130), which appended an 81-bp polyadenine tail to the transcription terminator [11]. Microinjection (MI) of two gRNAs (25 ng/μl each), Cas9-polyA mRNA (50 ng/l), and lssDNA (25 ng/μl) into 371 fertilized eggs from C57BL/6 mice resulted in 56 live births delivered from foster ICR mice (Table 1). PCR analysis of their tail tips using external primers outside HA sequences (Additional file 2: Figure S2) showed multiple bands, which appeared as mosaicism (more than three bands) in pups (Fig. 1b). Direct sequencing analysis of the PCR products demonstrated various insertions or deletions (indels) as well as large deletions (LDs) or inversions between the two cutting sites (Fig. 1c and Additional file 3: Figure S3). We found 16 pups carried loxP sequences, of which nine had two loxP sites floxing exon 4 (Table 1 and Fig. 1c). Among the 9 floxed mice (F0), 5 carried either homozygous floxed alleles or were heterozygous floxed with LD alleles, indicated as ‘Conditional’ knockouts in Table 1.

**Table 1:** CRISPR/Cas-mediated insertion of loxP sequences at targeted sites in mice and rats

Species	Strain	Target gene	lssDNA size (bp)	Transfer method	Embryos injected (n)	Embryos transferred (%)	Live births (%)	F0 animals			
								LD (%)	loxP (%)	flox (%)	Conditional (%)
Mouse	C57B6	*Serpina3n*	708	MI	371	255 (68.7)	56 (22.0)	28 (50.0)	7 (12.5)	9 (16.1)	5 (8.9)
	C57B6	*Tyr*	892	MI	222	149 (67.1)	17 (11.4)	2 (11.8)	4 (23.5)	3 (17.6)	–
	C57B6	*mKIAA1322*	1429	MI	166	134 (80.7)	5 (3.7)	1 (20.0)	–	4 (80.0)	2 (40.0)
	C57B6	*Serpina3n*	708	EL	180	160 (88.9)	18 (11.3)	9 (50.0)	2 (11.1)	2 (11.1)	2^b^ (11.1)
	C57B6	*Mct4*	1095	EL	134	130 (97.0)	21 (16.2)	11 (52.4)	3 (14.3)	2 (9.5)	1 (4.7)
Rat	F344	*Vapb*	674	EL	160	77 (48.1)	6 (7.8)	2 (33.3)	1 (16.6)	3^a^ (50.0)	1 (16.6)
Mouse	Emx1-cre	*Serpina3n*	708	MI	150	74 (49.3)	8 (10.8)	4 (50.0)	1 (12.5)	1 (12.5)	–
	Emx1-cre	*Serpina3n*	708	EL	150	113 (75.3)	27 (23.9)	10 (37.0)	1 (3.7)	5 (18.5)	3^c^ (11.1)

Transfer method: gRNA/Cas9 and long single-stranded DNA (lssDNA) transferred by microinjection (MI) or electroporation (EL). Embryos transferred: two-cell embryos were transferred into a surrogate mother. LD: large fragments deleted between two gRNA targeting sites. loxP and flox: positive for either one or two loxP sites, respectively

^a^All three rats carried a missense mutation, P56S, together with floxed alleles. Conditional: conditional knockouts by homozygous floxed alleles or heterozygous floxed with LD alleles in F0 animals

^b^Site-specific recombination confirmed by crossing with Cre-driver mice

^c^Site-specific recombination confirmed in Cre-expressing tissues of F0 mice

- not identified

This method was repeated for exon 2 of the Tyrosinase (*Tyr*) gene and for exon 3 of the *mKIAA1322* gene, which resulted in 3 mice carrying a floxed allele among 17 live births and 4 mice carrying a floxed allele among 5 live births, respectively (Table 1, Additional file 4: Figure S4, Additional file 5: Figure S5, Additional file 6: Figure S6, and Additional file 7: Figure S7). Crossing several F0 mice (#3, #22, #23 for floxed allele and #15, #20, #28 for single loxP-allele) with B6 mice confirmed the germline transmission of the floxed alleles into the next generation (Additional file 8: Figure 8). We observed no insertions or deletions at any off-target site for gRNAs targeting *Serpina3n* and *Tyr* genes across the whole genome in the F0 mice (Additional file 9: Figure 9).

### Floxed alleles generated by zygote electroporation in mice and rats

**Fig. 2:**
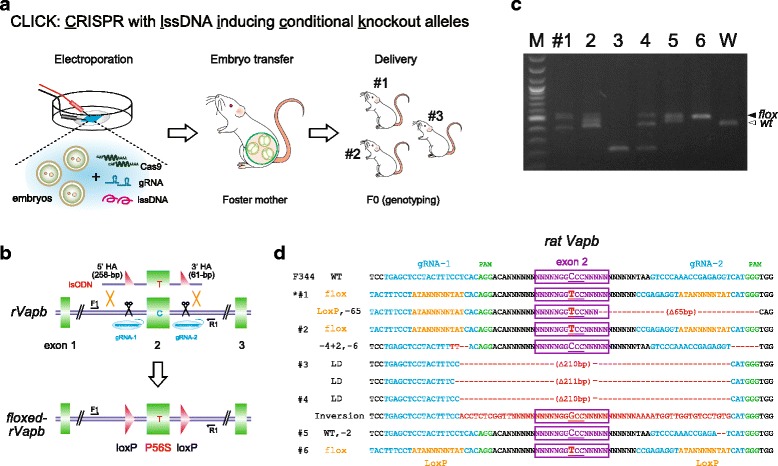
Rat floxed alleles generated by zygote electroporation. **a** Schematic representation of CLICK: CRISPR with lssDNA inducing conditional knockout alleles. **b** Schematic approach to generate rat Vapb floxed alleles using the CRISPR/Cas system with lssDNA composed of the targeted exon flanked by two loxP sites (Additional file 11: Figure S11). **c** PCR analysis of rat pups (#1–6) generated by CLICK, showing different types of mutations, indels, LD (via NHEJ), inversions, and floxed alleles (via HDR) (black arrow). The primer sets were used for PCR and sequence analysis (Additional file 2: Figure S2). M: 100 bp DNA ladder marker. **d** Sequence analysis of 6 pups showing a variety of mutations, indels, inversion or LD indicated by red letters, and loxP insertions and flox indicated by orange letters. All pups were used for testing germline transmission (Additional file 15: Table S1). Asterisks indicate pups used for testing the Cre-loxP system (Additional file 17: Table S3)

We have previously reported the easy and efficient generation of knockout (KO) or knock-in (KI) mice using the CRISPR/Cas9 system with zygote electroporation (EL) instead of MI [12, 13]. For KI of floxed alleles, we used lssDNA with CRISPR/Cas9 for the transfer into embryos by electroporation. The zygote EL of two gRNAs (100 ng/μl each), Cas9 mRNA (400 ng/μl), and lssDNA (40 ng/μl) for *Serpina3n* or Monocarboxylate transporter 4 (*Mct4*) genes into 180 and 134 fertilized eggs resulted in 18 and 21 live births, respectively (Table 1). PCR and sequence analysis demonstrated carriers with various mutations, indels, LDs, and floxed alleles (Additional file 10: Figure S10, Additional file 11: Figure S11 and Additional file 12: Figure S12). The live birth rate, indel mutation rate, and loxP insertion efficiency were similar to those of MI, except for the higher survival rates of two-cell embryos (80–90%) by EL, as previously reported [12, 13] (Table 1). Moreover, crossing the floxed mice with Cre-driver mice (B6.Cg-Tg(CAG-Cre)CZ-MO2Osb: RBRC01828) provided site-specific recombination events between the two loxP sites, which confirmed that the floxed alleles were functional (Additional file 13: Figure S13). Therefore, we named this method CLICK: CRISPR with lssDNA inducing conditional knockout alleles (Fig. 2a).

To test whether CLICK can be applied to other species such as rats, we designed lssDNA including two loxP sequences floxing exon 2 of the rat vesicle-associated membrane protein-associated protein B/C (*Vapb*) gene with a P56S mutation, which is associated with amyotrophic lateral sclerosis in humans [14] (Fig. 2b and Additional file 14: Figure S14). EL of the lssDNA and CRISPR components into rat zygotes resulted in the birth of six pups, which contained indels, LD, and inversion mutations between the cut sites (Table 1, Fig. 2c and d). Rats #1, #2, and #6 showed floxed alleles with the P56S mutation. Crossing these F0 rats with F344 rats or Cre-driver rats (W-Tg(CAG-cre)81Jmsk (NBRP-Rat No.0283)) provided the transmission of the floxed alleles or site-specific recombination that removed exon 2, respectively (Additional file 8: Figure S8 and Additional file 13: Figure S13).

### One-step generation of conditional knockout animals in the founder generation

**Fig. 3:**
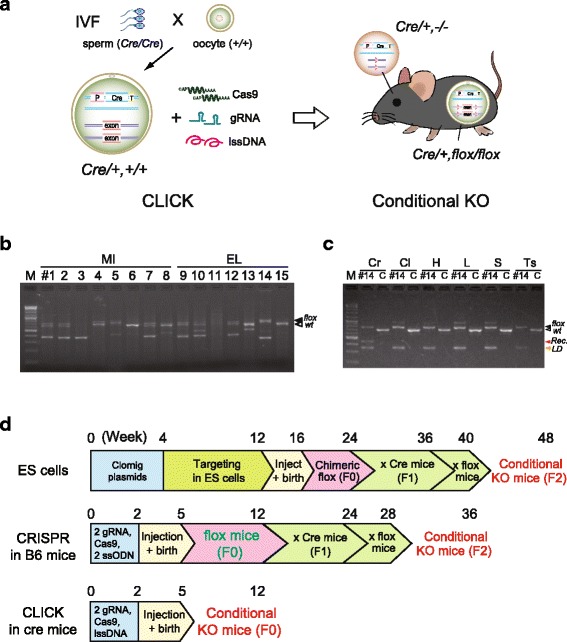
One-step generation of conditional knockout animals (F0) by CLICK. **a** Schematic representation of applying CLICK in oocytes for in vitro fertilization (IVF) with Cre-driver mice, Emx1-cre, resulting in brain-specific recombination at the targeted floxed alleles. **b** PCR analysis of representative delivered mouse pups by microinjection (#1–8) or electroporation (#9–15) showing different types of mutations, indels, LD, and floxed alleles (black arrow) at the targeted *Serpina3n* locus. The primer sets (‘small’ in Additional file 2: Figure S2) were used for PCR and sequence analysis. **c** Genotyping in several tissues: cerebrum (Cr), cerebellum (Cl), heart (H), liver (L), spleen (S), and testis (Ts), indicating recombination (red arrow) by brain-specific Cre expression in #14 mouse carrying heterozygous floxed with LD alleles. M: 100 bp DNA ladder marker. **d** Time-schedule comparisons of targeting methods using ES cells by CRISPR in B6 oocytes, and CLICK in Cre oocytes. CLICK saves about 6 months of crossing and reduces breeding costs during the study period (Additional file 13: Figure S13)

Finally, we applied the CLICK method to in vitro fertilized eggs of B6 mice with the sperm of Cre-driver mice (Emx1-cre: RBRC00808) expressing Cre in the cortical neurons and glia [15] (Fig. 3a). Among 8 and 27 live births by MI and EL, respectively, 6 mice carried floxed alleles for exon 2 of the *Serpina3n* gene (Table 1, Fig. 3b and Additional file 15: Table S1). Interestingly, 3 floxed mice, #14, #16, and #24, carried homozygous floxed alleles or heterozygous floxed with LD alleles in addition to the Emx1-Cre allele. PCR analysis of multiple tissues of #14 indicated a brain-specific recombination that removed exon 2 of the *Serpina3n* gene in this animal (Fig. 3c). These results indicate that CLICK can be used for the one-step generation of conditional knockout mice (F0) without further crossing, using fertilized eggs expressing tissue-specific Cre (Fig. 3d and Additional file 16: Table S2).

## Discussion

**Fig. 4:**
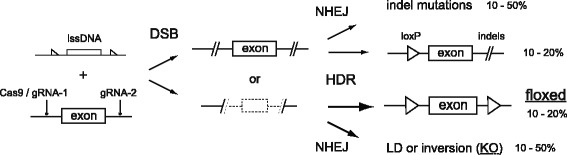
Schematic representation of various mutations generated by CLICK. Two gRNAs and Cas9 formed double-strand breaks (DSB) on each targeted site or a large deletion between the two sites. DSBs repaired by NHEJ caused indel mutations or LD. DSBs elicited a single loxP insertion or floxed allele by HDR repair

Compared with MI, CLICK with EL was less time-consuming, easier to prepare, and highly efficient at targeting the generation of floxed alleles in zygotes. lssDNA was easily prepared from custom-made plasmids within one day (Additional file 1: Figure S1). Zygote EL does not require expensive micromanipulator systems with special injection skills, allowing this approach to be used in numerous non-specialized laboratories (Fig. 2a). Frozen embryos or in vitro fertilized eggs are also available for zygote EL, facilitating the high throughput gene targeting of multiple genes with low costs (Fig. 3a). F0 mice generated by CLICK showed both homozygous floxed alleles and heterozygous or mosaic alleles, which in most cases were passed to the offspring (Additional file 15: Table S1). In principal, two gRNA and Cas9 transferred into zygotes provided double-strand breaks (DSBs) on each targeted site (Fig. 4). These DSBs were repaired by NHEJ, causing indel mutations at each site or a LD mutation. DSBs repaired by HDR using the lssDNA elicited a single loxP insertion or a floxed allele. Our data indicated a positive correlation between the observed number of large deletions and the occurrence of floxed alleles. To generate more LDs, three or four gRNAs may be used as previously reported [16, 17].

Although random integrations of single-stranded DNA are an infrequent event in contrast to those of double-stranded DNA, NHEJ-mediated mutations or DNA insertions at off-target sites may eventually occur. In the F0 founders we tested, no off-target insertion of the lssDNA donors was observed or determined by checking the heritability of the targeted floxed alleles in F1 mice. However, further backcrossing to wild-type animals might segregate such off-target integrations if detected.

We previously reported lssDNA, formerly named lsODN, for CRISPR-mediated efficient KIs of GFP-coding sequences in rat zygotes [11]. CLICK with lssDNA including Cre-coding sequences in mouse zygotes also provided efficient KIs of Cre alleles at an endogenous targeted gene (T Mashimo, pers. comm.). Recently, Quadros et al. reported highly efficient KIs of Cre or floxed alleles (about 10–100%) using the microinjection of Cas9 protein with lssDNA into mouse embryos [18, 19]. However, Remy et al. reported no integration of a GFP reporter cassette with lssDNA by zygote electroporation, although the large DNA fragment was transferred into embryos [20]. The three different protocols used for the preparation of lssDNAs [11, 19, 20] might result in the different efficiency of KIs. According to our reports [11] and a previous report [19], lssDNA generally provides more efficient KIs of approximately 10–50% in zygotes compared with double stranded DNA that normally provides less than 10%. In addition, Cas9 protein rather than Cas9 mRNA provides more efficient KIs with short or long ssDNAs. The limitations of the current lssDNA approaches are the maximal length of the synthetic lssDNA is up to 3 kb, although this size is sufficient to generate floxed alleles for a single exon of most genes.

The novel aspect of this study was the zygote electroporation of lssDNA from 600 to 1.5 kb to generate floxed alleles. In addition, CLICK in oocytes carrying tissue-specific Cre allowed the one-step generation of ‘conditional knockouts’ in F0 founder mice. In this study, F0 floxed mice showed brain-specific recombination that removed exon 2 of the *Serpina3n* gene (Fig. 3c). Immunostaining of brains with an anti-Serpina3n antibody showed the reduced expression of Serpina3n protein in F0 mice compared with wild-type mice (Additional file 17: Table S3). To the best of our knowledge, this one-step generation of conditional knockout mice has not been reported previously. This method could be used to generate conditional knockout mice routinely. However, the potential risk of mosaicism should be characterized in F0 mice. The conditional knockout phenotypes must be reconfirmed in the cohorts of the next F1 generation to distinguish tissue specific recombination or the extent of mosaicism. If the germline of F0 mice is not recombined by Cre expression, conditional knockout phenotypes could be easily reconfirmed in the next generation.

## Conclusions

In summary, the use of CLICK in the zygote electroporation of two gRNAs, Cas9 and lssDNA, provides the easy and quick generation of floxed alleles in zygotes (Fig. 3d). Furthermore, CLICK in fertilized oocytes expressing tissue-specific Cre enables the one-step generation of conditional knockout mice, skipping two generations for subsequent crossing, which facilitates high-throughput gene targeting in mice and rats.

## Methods

### Animals

C57BL/6JJcl mice and F344/Jcl rats were obtained from CLEA Japan Inc., Tokyo, Japan. B6.Cg-Tg(CAG-Cre)CZ-MO2Osb (RBRC01828) and Emx1-cre (RBRC00808) mice were provided by RIKEN BRC (www.en.brc.riken.jp), and W-Tg(CAG-cre)81Jmsk (NBRP-Rat No.0283) rats were from the National Bio Resource Project for the Rat in Japan (www.anim.med.kyoto-u.ac.jp/nbr). The animals were kept under conditions of 50% humidity and a 12:12 h light:dark cycle. They were fed a standard pellet diet (MF, Oriental Yeast Co., Tokyo, Japan) and tap water *ad libitum*.

### Preparation of Cas9, gRNA and lssDNAs

We formally constructed pCas9-polyA and deposited it into the Addgene repository (ID #72602; www.addgene.org/CRISPR). mRNA was transcribed in vitro using a mMESSAGE mMACHINE T7 Ultra Kit (Life Technologies, Carlsbad, CA, USA) from linearized plasmids and was purified using a MEGAClear kit (Life Technologies). To design gRNAs, software tools (www.crispr.genome-engineering.org) predicting unique target sites throughout the mouse and rat genome were used. gRNAs were transcribed in vitro using a MEGAshortscript T7 Transcription Kit (Life Technologies) from synthetic double-strand DNAs obtained from IDT (Integrated DNA Technologies, IA, USA) or Life Technologies.

lssDNAs were prepared by a simple method using nicking endonucleases as we previously reported [11] (Additional file 1: Figure S1). Briefly, double-stranded DNA plasmids comprising of a floxed allele, homology arms, and two nicking endonuclease sites were obtained from Thermo Fisher Scientific (MA, USA) as GeneArt^®^ Gene Synthesis. For digestion, 100 μg of the purified plasmid DNA was incubated at an optimum temperature for 2 to 3 h with nicking endonucleases, such as Nt.*BspQI* and Nb.*BbvCI* (New England Biolabs Inc., MA, USA). After purification by ethanol precipitation, the DNAs were denatured with 3-fold amounts of formamide (Nacalai Tesque, Inc., Tokyo, Japan) at 80 °C for 10 min, and then subjected to agarose gel electrophoresis with DynaMarker^®^Prestain Marker for RNA High concentrations (Biodynamics Laboratory Inc., Tokyo, Japan). Bands corresponding to a single-strand DNA fragment were extracted using NucleoSpin^®^ Gel and PCR Clean-up (Takara Bio, Shiga, Japan). Finally, 2–4 μg of lssDNA was obtained with this method.

### Microinjection and electroporation into mouse and rat embryos

Pronuclear-stage mouse embryos were prepared by thawing frozen embryos (CLEA Japan Inc.), or in vitro fertilized embryos, or fresh embryos collected from naturally mated female mice that were superovulated by injection with pregnant mare serum gonadotropin (PMSG: ASKA Animal Health Co., Tokyo, Japan) and human chorionic gonadotropin (HCG: ASKA Animal Health Co.). Rat embryos were collected from 8 to 12 weeks of age females that were superovulated by the administration of 150 U/kg of PMSG followed by 75 U/kg of HCG. After natural mating, pronuclear-stage embryos were collected from the oviducts of the females and cultured in a modified Krebs–Ringer bicarbonate medium or KSOM medium (ARK Resource, Kumamoto, Japan).

In MI, 50 ng/μl Cas9 mRNA, 25 ng/μl gRNA, or 50 ng/μl lssDNA were microinjected into the male pronuclei of embryos using a micromanipulator (Narishige, Tokyo, Japan). In targeting mouse *KIAA1322*, 30 ng/μl Cas9 protein (PNA Bio, Thousand Oaks, CA, USA) was used instead of Cas9 mRNA [21]. This was cultured in modified Krebs–Ringer bicarbonate or KSOM medium overnight and divided two-cell embryos were transferred into pseudopregnant females.

For EL, 50–100 embryos at 1 h after thawing or 3–4 h after collection were placed into a chamber with 40 μl of serum free media (Opti-MEM, Thermo Fisher Scientific) containing 400 ng/μl Cas9 mRNA, 200 ng/μl gRNA, or 40 ng/μl lssDNA. They were electroporated with a 5 mm gap electrode (CUY505P5 or CUY520P5 Nepa Gene, Chiba, Japan) in a NEPA21 Super Electroporator (Nepa Gene, Chiba, Japan).The poring pulses for the electroporation were voltage 225 V, pulse width 1.5 ms for mouse embryos and 2.0 ms for rat embryos, pulse interval 50 ms, and number of pulses 4. The first and second transfer pulses were voltage 20 V, pulse width 50 ms, pulse interval 50 ms, and number of pulses 5. Mouse or rat embryos that developed to the two-cell stage after the introduction of RNA and lssDNA were transferred into the oviducts of female surrogates anesthetized with isoflurane (DS Pharma Animal Health Co., Ltd., Osaka, Japan).

### Genotyping analysis

Genomic DNA was extracted from the tail tip using the KAPA Express Extract DNA Extraction Kit (Kapa Biosystems, London, UK). For PCR and sequence analysis, we used external primers outside the HA, which amplied the targeted region (Additional file 18: Table S4). PCR was performed in a total volume of 15 μl under the following conditions: 1 cycle of 94 °C for 3 min; 35 cycles of 94 °C for 30 s, 60 °C for 1 min and 72 °C for 45 s; and 1 cycle of 72 °C for 3 min. The final reaction mixture contained 200 μM dNTPs, 1.0 mM MgCl_2_, and 0.66 μM of primer. The PCR products were then directly sequenced using the BigDye Terminator v3.1 cycle sequencing mix and the standard protocol for an Applied Biosystems 3130 DNA Sequencer (Life Technologies). To confirm mosaic mutations, we sequenced individual TA clones in some cases.

## Additional files

Additional file 1: **Figure S1.** Schematic representation of the protocol to prepare long single-stranded DNA (lssDNA) using nicking endonucleases. (AI 1147 kb)

Additional file 2: **Figure S2.** Sequences of two gRNAs and a complementary lssDNA(−) designed to target exon 4 of the mouse *Serpina3n* locus. (AI 1215 kb)

Additional file 3: **Figure S3.** Floxed *Serpina3n* alleles generated by the microinjection of two gRNA, Cas9 mRNA and lssDNA(−) into B6 mouse zygotes. (AI 1263 kb)

Additional file 4: **Figure S4.** Sequences of two gRNAs and lssDNA(−) designed to target exon 2 of the mouse *Tyr* locus. (AI 1210 kb)

Additional file 5: **Figure S5.** Floxed *Tyr* alleles generated by the microinjection of two gRNA, Cas9 mRNA and lssDNA(−) into B6 mouse zygotes. (AI 1656 kb)

Additional file 6: **Figure S6.** Sequences of two gRNAs and lssDNA(−) designed to target exon 3 of the mouse *KIAA1322* locus. (AI 1214 kb)

Additional file 7: **Figure S7.** Floxed *KIAA1322* alleles generated by the microinjection of two gRNA, Cas9 mRNA and lssDNA(−) into B6 mouse zygotes. (AI 2040 kb)

Additional file 8: **Figure S8.** Floxed *Serpina3n* alleles generated by the electroporation of two gRNA, Cas9 mRNA and lssDNA(−) into B6 mouse zygotes. (AI 1569 kb)

Additional file 9: **Figure S9.** Sequences of two gRNAs and lssDNA(+) designed to target exon 3 of the mouse *Mct4* locus. (AI 1209 kb)

Additional file 10: **Figure S10.** Floxed *Mct4* alleles generated by the electroporation of two gRNA, Cas9 mRNA and lssDNA(+) into B6 mouse zygotes. (AI 1651 kb)

Additional file 11: **Figure S11.** Sequences of two gRNAs and lssDNA(+) designed to target exon 2 of the rat *Vapb* locus. (AI 1013 kb)

Additional file 12: **Figure S12.** Floxed *Serpina3n* alleles generated by CLICK in oocytes in vitro fertilized with the sperm of Emx1-Cre driver mice. (AI 1260 kb)

Additional file 13: **Figure S13.** Schematic representation of the one-step generation of conditional knockout animals (F0) by CLICK. (AI 1315 kb)

Additional file 14: **Figure S14.** Expression levels of SERPINA3N protein in the brains of wild-type (WT) and F0 founder mice (#14). (AI 1698 kb)

Additional file 15: **Table S1.** Germline transmission of CLICK-mediated mutations in F1 offspring. (XLSX 12 kb)

Additional file 16: **Table S2.** Potential off-target sites for CRISPR/Cas targeting on the mouse genome. (XLSX 14 kb)

Additional file 17: **Table S3.** Crossing floxed founders with Cre-driver mice. (XLSX 11 kb)

Additional file 18: **Table S4.** Primer sets used for PCR and sequencing analysis in this study. (XLSX 10 kb)

## Abbreviations

*CLICK*: CRISPR with lssDNA inducing conditional knockout
*CRISPR*: clustered regularly interspaced short palindromic repeats
*EL*: Electroporation
*HA*: Homology arm
*HDR*: Homology-directed repair
*KI*: Knock-in
*KO*: Knockout
*LD*: Large deletion
*lssDNA*: Long single-stranded DNA
*MI*: Microinjection
*NHEJ*: Non-homologous end-joining
*ssODN*: Single-stranded oligodeoxynucleotide

## References

[1] Lobe CG, Nagy A (1998). Conditional genome alteration in mice. BioEssays.

[2] Skarnes WC, Rosen B, West AP, Koutsourakis M, Bushell W, Iyer V, Mujica AO, Thomas M, Harrow J, Cox T (2011). A conditional knockout resource for the genome-wide study of mouse gene function. Nature.

[3] Friedel RH, Seisenberger C, Kaloff C, Wurst W (2007). EUCOMM--the European conditional mouse mutagenesis program. Brief Funct Genomic Proteomic.

[4] Lloyd KC (2011). A knockout mouse resource for the biomedical research community. Ann N Y Acad Sci.

[5] Wang H, Yang H, Shivalila CS, Dawlaty MM, Cheng AW, Zhang F, Jaenisch R (2013). One-step generation of mice carrying mutations in multiple genes by CRISPR/Cas-mediated genome engineering. Cell.

[6] Yang H, Wang H, Shivalila CS, Cheng AW, Shi L, Jaenisch R (2013). One-step generation of mice carrying reporter and conditional alleles by CRISPR/Cas-mediated genome engineering. Cell.

[7] Li D, Qiu Z, Shao Y, Chen Y, Guan Y, Liu M, Li Y, Gao N, Wang L, Lu X (2013). Heritable gene targeting in the mouse and rat using a CRISPR-Cas system. Nat Biotechnol.

[8] Brown AJ, Fisher DA, Kouranova E, McCoy A, Forbes K, Wu Y, Henry R, Ji D, Chambers A, Warren J (2013). Whole-rat conditional gene knockout via genome editing. Nat Methods.

[9] Bishop KA, Harrington A, Kouranova E, Weinstein EJ, Rosen CJ, Cui X, Liaw L. CRISPR/Cas9 mediated insertion of loxP sites in the mouse Dock7 gene provides an effective alternative to use of targeted embryonic stem cells. G3 (Bethesda). 2016; 10.1534/g3.116.030601 PMC4938658 27175020

[10] Ma Y, Zhang X, Shen B, Lu Y, Chen W, Ma J, Bai L, Huang X, Zhang L (2014). Generating rats with conditional alleles using CRISPR/Cas9. Cell Res.

[11] Yoshimi K, Kunihiro Y, Kaneko T, Nagahora H, Voigt B, Mashimo T (2016). ssODN-mediated knock-in with CRISPR-Cas for large genomic regions in zygotes. Nat Commun.

[12] Kaneko T, Sakuma T, Yamamoto T, Mashimo T (2014). Simple knockout by electroporation of engineered endonucleases into intact rat embryos. Sci Rep.

[13] Qin W, Dion SL, Kutny PM, Zhang Y, Cheng AW, Jillette NL, Malhotra A, Geurts AM, Chen YG, Wang H (2015). Efficient CRISPR/Cas9-mediated genome editing in mice by zygote electroporation of nuclease. Genetics.

[14] Nishimura AL, Mitne-Neto M, Silva HC, Richieri-Costa A, Middleton S, Cascio D, Kok F, Oliveira JR, Gillingwater T, Webb J (2004). A mutation in the vesicle-trafficking protein VAPB causes late-onset spinal muscular atrophy and amyotrophic lateral sclerosis. Am J Hum Genet.

[15] Iwasato T, Nomura R, Ando R, Ikeda T, Tanaka M, Itohara S (2004). Dorsal telencephalon-specific expression of Cre recombinase in PAC transgenic mice. Genesis.

[16] Essletzbichler P, Konopka T, Santoro F, Chen D, Gapp BV, Kralovics R, Brummelkamp TR, Nijman SM, Burckstummer T (2014). Megabase-scale deletion using CRISPR/Cas9 to generate a fully haploid human cell line. Genome Res.

[17] Zhou H, Liu B, Weeks DP, Spalding MH, Yang B (2014). Large chromosomal deletions and heritable small genetic changes induced by CRISPR/Cas9 in rice. Nucleic Acids Res.

[18] Miura H, Gurumurthy CB, Sato T, Sato M, Ohtsuka M (2015). CRISPR/Cas9-based generation of knockdown mice by intronic insertion of artificial microRNA using longer single-stranded DNA. Sci Rep.

[19] Quadros RM, Miura H, Harms DW, Akatsuka H, Sato T, Aida T, Redder R, Richardson GP, Inagaki Y, Sakai D (2017). Easi-CRISPR: a robust method for one-step generation of mice carrying conditional and insertion alleles using long ssDNA donors and CRISPR ribonucleoproteins. Genome Biol.

[20] Remy S, Chenouard V, Tesson L, Usal C, Menoret S, Brusselle L, Heslan JM, Nguyen TH, Bellien J, Merot J (2017). Generation of gene-edited rats by delivery of CRISPR/Cas9 protein and donor DNA into intact zygotes using electroporation. Sci Rep.

[21] Aida T, Chiyo K, Usami T, Ishikubo H, Imahashi R, Wada Y, Tanaka KF, Sakuma T, Yamamoto T, Tanaka K (2015). Cloning-free CRISPR/Cas system facilitates functional cassette knock-in in mice. Genome Biol.

